# There is variability in the attainment of developmental milestones in the CDKL5 disorder

**DOI:** 10.1186/1866-1955-7-2

**Published:** 2015-01-05

**Authors:** Stephanie Fehr, Helen Leonard, Gladys Ho, Simon Williams, Nick de Klerk, David Forbes, John Christodoulou, Jenny Downs

**Affiliations:** Telethon Kids Institute, University of Western Australia, Perth, Western Australia Australia; School of Physiotherapy and Exercise Science, Curtin University, Perth, Western Australia Australia; Western Sydney Genetics Program, Children’s Hospital at Westmead, Sydney, NSW Australia; Disciplines of Paediatrics & Child Health and Genetic Medicine, University of Sydney, Sydney, NSW Australia; Department of Neurology and Rehabilitation, Princess Margaret Hospital, Perth, Western Australia Australia; School of Paediatrics and Child Health, University of Western Australia, Perth, Western Australia Australia

**Keywords:** CDKL5 disorder, Developmental disabilities, Epileptic encephalopathy, Early infantile epileptic encephalopathy

## Abstract

**Background:**

Individuals with the CDKL5 disorder have been described as having severely impaired development. A few individuals have been reported having attained more milestones including walking and running. Our aim was to investigate variation in attainment of developmental milestones and associations with underlying genotype.

**Methods:**

Data was sourced from the International CDKL5 Disorder Database, and individuals were included if they had a pathogenic or probably pathogenic *CDKL5* mutation and information on early development. Kaplan-Meier time-to-event analyses investigated the occurrence of developmental milestones. Mutations were grouped by their structural/functional consequence, and Cox regression was used to investigate the relationship between genotype and milestone attainment.

**Results:**

The study included 109 females and 18 males. By 5 years of age, only 75% of the females had attained independent sitting and 25% independent walking whilst a quarter of the males could sit independently by 1 year 3 months. Only one boy could walk independently. No clear relationship between mutation group and milestone attainment was present, although females with a late truncating mutation attained the most milestones.

**Conclusion:**

Attainment of developmental milestones is severely impaired in the CDKL5 disorder, with the majority who did attain skills attaining them at a late age. It appears as though males are more severely impaired than the females. Larger studies are needed to further investigate the role of genotype on clinical variability.

## Background

The majority of research on individuals with mutations in the cyclin-dependent kinase-like 5 (*CDKL5*) gene has been limited to case studies and small case series (*n* = 20) [1–3]. These studies have predominantly focused on individuals with the early-onset seizure variant of Rett syndrome (RTT). However, using information collected through the International Rett Syndrome Phenotype Database (InterRett) [4], we found that only a small proportion of individuals with a *CDKL5* mutation met the clinical criteria for the early-onset seizure variant of RTT, which specifically requires a period of developmental regression [5]. We therefore suggested that the term ‘CDKL5 disorder’ was a more appropriate terminology for individuals with a mutation in the *CDKL5* gene.

The literature suggests that individuals with the CDKL5 disorder rarely experience normal development and that the majority have refractory epilepsy with onset within the first few months of life and severe global developmental delay [6–8]. There has however been a small proportion reported with the ability to walk and even to run [9–12], suggesting that the clinical severity may be more variable than originally thought. Variability in clinical presentation is also seen in RTT, which is mostly associated with mutations in the methyl-CpG binding domain-2 gene (*MECP2*) [5]. This clinical variability has resulted in ‘variant’ types being described and studies focusing on investigating the relationship between clinical presentation and specific mutations [13–15]. For caregivers and clinicians, it is important that variability in the CDKL5 disorder be further examined so that appropriate prognostic information can be provided. Further investigation is needed to develop a better clinical overview of the development and attainment of milestones in this disorder. Our aim was to describe the attainment of gross motor, hand function, communication and other developmental milestones in individuals with the CDKL5 disorder. We also investigated the relationship between the timing of these events and genotype.

## Methods

**Figure 1:**
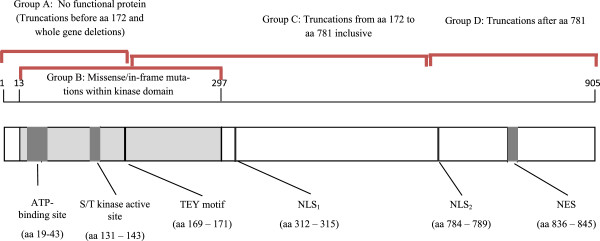
**Schematic representation of the CDKL5 protein and our mutational grouping.** Legend: The catalytic region is shown in light grey and the C-terminal region in white. Modified from ‘*What We Know and Would Like to Know about CDKL5 and its Involvement in Epileptic Encephalopathy*’ [17]. *TEY motif* Thr-Glu-Tyr motif, *NLS*_*1*_ and *NLS*_*2*_ nuclear localisation signals 1 and 2, *NES* nuclear export signal.

Information on individuals with the CDKL5 disorder was obtained from the newly developed International CDKL5 Disorder Database, which was established in September 2012. Data is collected from caregivers in the form of online or paper-based questionnaires. Caregivers were asked to refer to their child’s infant health record, diaries or available medical records when completing the questionnaire. Cases were included in this study if the individuals *CDKL5* mutation was considered to be pathogenic or probably pathogenic, and the caregiver had completed the questionnaire section on early development. Due to the heterogeneity of mutations within our sample, each individual’s mutation was grouped according to its predicted structural/functional consequence [16]. These groups were a) mutations causing no functional protein which included mutations resulting in a loss of the functional components in the catalytic domain (portion of protein responsible for kinase activity) before amino acid (aa) 172 and all full gene deletions, b) missense/in-frame mutations within the catalytic domain which included any missense mutations within the protein kinase active region or in-frame mutations (such as a deletion resulting in loss of some of the kinase region but subsequent protein is intact), c) truncating mutations located from aa 172 to aa 781 inclusive which includes any mutations resulting in a truncation such as nonsense or frameshift mutations resulting in maintaining kinase activity but loss of c-terminal region and d) truncating mutations occurring after aa 781 and therefore maintaining kinase activity and majority of the c-terminal region (Figure 1).

To investigate early development, we included information on the age of attainment of the following major developmental milestones: sitting, standing and walking independently; raking grasp and pincer grip; and babble and use of single words. The timing of attainment of these milestones was investigated using Kaplan-Meier time-to-event analysis. This allows an appropriate estimate of the proportion of individuals who will attain a skill by a particular age whilst taking into account those who are still yet to attain the skill. To investigate the relationship between mutation type and the attainment of these particular milestones, separate time-to-event analyses by mutation group were undertaken and the median age and proportion that attained each skill determined. Cox regression was used to investigate the relationship between each skill and mutation group.

Information on the attainment of additional milestones, for which we did not collect an age of attainment, was also included. For these skills, individuals were grouped according to their current age at time questionnaire completion (younger than 1.5 years; 1.5 to <7 years; 7 to <13 years; and 13 years and older) and we determined the proportion of each age group who had attained the milestone of interest. These age groups were chosen based on the expected developmental level of the children. All the milestones included in our study (excluding the use of phrases) would have been achieved by 1.5 years of age in a normally developing child. We therefore grouped those aged 1.5 years and under as it was likely that these children were yet to attain many of these skills. This allowed us to account for young individuals who had not yet had the opportunity to attain these skills.

Approval for this study was provided by the University of Western Australia Human Research Ethics Committee, Perth, Western Australia, Australia (RA/4/1/5024).

## Results

As of May 2014, there were 127 individuals for whom information on early development had been provided. Of these, 109 (86%) were female (median age 6.1 years, range 3 months to 29 years) and 18 (14%) were male (median age 4.6 years, range 10 months to 22.7 years). The distribution of mutations within our sample is shown in Table 1. We have also included information on our determination of whether missense mutations were likely pathogenic (Table 2).

**Table 1:** **Distribution of** ***CDKL5*** **mutations within our study group**

Mutation group	Nucleotide change (cDNA)	Protein change	Exon/intron	Individuals (gender)	Reported mutation	Reported individual
No functional protein	c.-253-?_-163 + ?del	p.(0)	1	2 (F)	Yes	Yes [4]
No functional protein	c.-253-?_-163 + ?del	p.(0?)	1	1 (F)	Yes	Yes [4]
No functional protein	c.-253-?_99 + ?del	p.(0)	1–3	1 (F)	Yes	Yes [4]
No functional protein	c.64 + 2 T > C	p.(0)	2	1 (F)	Yes	No
No functional protein	c.64 + 1G > A	p.(0)	2	1 (F)	Yes	Yes [4]
No functional protein	c.-162-?_99 + ?del	p.(0)	2–3	1 (F)	Yes	Yes [4]
No functional protein	c.-162-?_99 + ?	p.(0)	2–3	1 (F)	No	No
No functional protein	c.-253-?_*1085del	p.(0)	1–21+	1 (F)	No	No
No functional protein	c.-253-?_*1085del	p.(0)	1–21	3(F)	No	No
No functional protein	c.-253-?_*1085del	p.(0)	1–21+	1 (F)	No	No
No functional protein	c.-253-?_*1085del	p.(0)	1–21+	1 (F)	Yes	Yes [4]
No functional protein	c.-253-?_*1085del	p.(0)	1–21+	1 (F)	No	No
No functional protein	c.65-?_99 + ?del	p.(Ala23Asnfs*3)	3	2 (F and M)	Yes	Yes [4]
No functional protein	c.99 + 5G > A	p.Ala23Asnfs*3	3	1 (F)	Yes	Yes [2, 4]
No functional protein	c.100-?_*1085del	p.(?)	4–21+	1 (F)	No	No
No functional protein	c.146-1G > A	p.(Asn50Tyrfs*15)	5	1 (M)	No	No
No functional protein	c.146-6 T > G	p.(Glu49Valfs*2)	5	1 (F)	No	No
No functional protein	c.146-?_282 + ?del	p.(Asn50Tyrfs*15)	5	1 (F)	Yes	Yes [4]
No functional protein	c.163_166delGAAA	p.Glu55Argfs*20	5	1 (F)	Yes	No
No functional protein	c.175C > T	p.Arg59*	5	5 (4 and 1 M)	Yes	Yes [4, 18, 19]
No functional protein	c.183delT	p.Met63Cysfs*13	5	2 (F, twins)	Yes	Yes [4]
No functional protein	c.220G > T	p.Glu74*	5	1 (F)	No	No
No functional protein	c.282 + 3_282 + 6delAAGT	p.(Asn50Tyrfs*15)	5	2 (F)	Yes	Yes [4]
No functional protein	c.283-3_290del	p.(Asn95Ilefs*2)	5	2 (F, sisters)	Yes	Yes [2, 4]
No functional protein	c.339ins1	p.?	6	1 (F)	No	No
No functional protein	c.351 T > A	p.Tyr117*	6	1 (M)	No	No
No functional protein	c.400C > T	p.Arg134*	6	2 (F and M)	Yes	Yes [4, 20]
No functional protein	c.456_457delTG	p.Cys152*	7	1 (F)	No	No
No functional protein	c.464-2A > G	p.Gly155Alafs*43	7	1 (M)	Yes	No
No functional protein	c.506_507delAC	p.Thr168Argfs*36	8	1 (F)	No	No
Missense/in-frame mutation within catalytic domain	c.38 T > C	p.Phe13Ser	2	1 (F)	Yes	Yes [4]
Missense/in-frame mutation within catalytic domain	c.58G > C	p.Gly20Arg	2	1 (F)	Yes	Yes [4, 8]
Missense/in-frame mutation within catalytic domain	c.100-9_100-3delCCCTTGCinsGCAGA	p.(Lys33dup)	4	1 (F)	No	No
Missense/in-frame mutation within catalytic domain	c.119C > T	p.Ala40Val	4	1 (F)	Yes	Yes [4]
Missense/in-frame mutation within catalytic domain	c.191 T > C	p.Leu64Pro	5	1 (M)	Yes	Yes [4, 21]
Missense/in-frame mutation within catalytic domain	c.214_216del	p. Ile72del	5	1 (F)	No	No
Missense/in-frame mutation within catalytic domain	c.215 T > C	p.Ile72Thr	5	1 (F)	Yes	No
Missense/in-frame mutation within catalytic domain	c.364G > A	p.Ala122Thr	6	1 (F)	Yes	Yes [4]
Missense/in-frame mutation within catalytic domain	c.377G > A	p.Cys126Tyr	6	1 (F)	Yes	Yes [4]
Missense/in-frame mutation within catalytic domain	c.395 T > G	p.Val132Gly	6	1 (F)	No	No
Missense/in-frame mutation within catalytic domain	c.404A > G	p.(Asp135_Phe154del)	7	1 (M)	No	No
Missense/in-frame mutation within catalytic domain	c.404-3C > A	p.(Asp135_Phe154del)	7	1 (F)	Yes	Yes [4]
Missense/in-frame mutation within catalytic domain	c.428 T > A	p. Ile143Asn	7	1 (F)	No	No
Missense/in-frame mutation within catalytic domain	c.458A > T	p. Asp153Val	7	1 (F)	No	No
Missense/in-frame mutation within catalytic domain	c.463 + 1G > A	p.Asp135_Phe154del	7	1 (F)	Yes	Yes [4]
Missense/in-frame mutation within catalytic domain	c.473G > C	p.Arg158Pro	8	1 (M)	Yes	Yes [4]
Missense/in-frame mutation within catalytic domain	c.514G > A	p.Val172Ile	8	1 (M)	No	No
Missense/in-frame mutation within catalytic domain	c.526 T > C	p.Trp176Arg	8	1 (F)	No	No
Missense/in-frame mutation within catalytic domain	c.532C > T	p.Arg178Trp	8	2 (F)	Yes	Yes [4]
Missense/in-frame mutation within catalytic domain	c.536C > T	p. Ser179Phe	8	1 (F)	No	No
Missense/in-frame mutation within catalytic domain	c.577G > C	p.Asp193His	9	1 (F)	No	No
Missense/in-frame mutation within catalytic domain	c.587C > T	p.Ser196Leu	9	5 (F)	Yes	Yes [4]
Missense/in-frame mutation within catalytic domain	c.595 T > C	p.Cys199Arg	9	1 (F)	Yes	Yes [4]
Missense/in-frame mutation within catalytic domain	c.620G > A	p.Gly207Glu	9	1 (F)	Yes	Yes [4]
Missense/in-frame mutation within catalytic domain	c.656A > C	p.Gln219Pro	9	1 (F)	Yes	Yes [4]
Missense/in-frame mutation within catalytic domain	c.680 T > C	p.Leu227Pro	9	1 (F)	Yes	Yes [4]
Missense/in-frame mutation within catalytic domain	c.872G > A	p.Cys291Tyr	11	1 (F)	Yes	No
Truncation occurring between aa 172 and 781	c.670C > T	p.Gln224*	9	1 (M)	No	No
Truncation occurring between aa 172 and 781	c.556_557delGC	p.Ala186Serfs*19	9	1 (M)	No	No
Truncation occurring between aa 172 and 781	c.745-?_977 + ?del	p.(Phe249Lysfs*16)	10–11	1 (F)	No	No
Truncation occurring between aa 172 and 781	c.801_802delAT	p.Asn267Lysfs*5	11	1 (F)	No	No
Truncation occurring between aa 172 and 781	c.857dupA	p.Tyr286*	11	1 (F)	Yes	Yes [4]
Truncation occurring between aa 172 and 781	c.859_868del10	p.Leu287Serfs*3	11	1 (F)	Yes	Yes [4]
Truncation occurring between aa 172 and 781	c.978-?_2980 + ?del	p.?	12+	1 (F)	No	No
Truncation occurring between aa 172 and 781	c.1039C > T	p.Gln347*	12	1 (F)	Yes	No
Truncation occurring between aa 172 and 781	c.1371dupA	p.Leu458Thrfs*5	12	1 (F)	Yes	Yes [4]
Truncation occurring between aa 172 and 781	c.1375C > T	p.Gln459*	12	1 (F)	Yes	Yes [4]
Truncation occurring between aa 172 and 781	c.1446delC	p.Tyr482*	12	1 (F)	Yes	Yes [4]
Truncation occurring between aa 172 and 781	c.1470_1471delGG	p.Ala491Thrfs*3	12	1 (F)	Yes	Yes [4]
Truncation occurring between aa 172 and 781	c.1499ins4	p.?	12	1 (F)	Yes	Yes [4]
Truncation occurring between aa 172 and 781	c.1547_1554del8	p.Tyr516Phefs*2	12	1 (F)	No	No
Truncation occurring between aa 172 and 781	c.1581del	p.Pro527fs	12	1 (F)	No	No
Truncation occurring between aa 172 and 781	c.1648C > T	p.Arg550*	12	4 (F)	Yes	Yes (1 child [4])
Truncation occurring between aa 172 and 781	c.1671dupA	p.Arg558Thrfs*9	12	1 (F)	Yes	No
Truncation occurring between aa 172 and 781	c.1675C > T	p.Arg559*	12	1 (F)	Yes	Yes [4]
Truncation occurring between aa 172 and 781	c.1782 T > G	p.Tyr594*	12	1 (F)	Yes	Yes [4]
Truncation occurring between aa 172 and 781	c.1791delC	p.Tyr598Thrfs*18	12	1 (F)	Yes	Yes [4]
Truncation occurring between aa 172 and 781	c.2038A > T	p.Lys680*	13	1 (F)	Yes	Yes [4]
Truncation occurring between aa 172 and 781	c.2047-1G > A	p.Gly683Valfs*101	13	1 (F)	Yes	Yes [4]
Truncation occurring between aa 172 and 781	c.2047-2A > G	p.Gly683Cysfs*66	13	1 (F)	Yes	Yes [4]
Truncation occurring between aa 172 and 781	c.2072_2073delCT	p.Ser691*	14	1 (F)	Yes	Yes [4]
Truncation occurring between aa 172 and 781	c.2258_2259delAA	p.Gln753Profs*10	15	1 (F)	No	No
Truncation occurring between aa 172 and 781	c.2376 + 5G > A	p.(Lys760Tyrfs*10)	16	1 (F)	Yes	Yes [4]
Late truncation after aa 781	c.2374dupA	p.Thr792Asnfs*9	16	1 (F)	Yes	Yes [4]
Late truncation after aa 781	c.2377-8 T > A	p.(Val793Leufs*2)	16	1 (F)	No	No
Late truncation after aa 781	c.2413C > T	p.Gln805*	17	1 (M)	No	No
Late truncation after aa 781	c.2420_2430del	p.Ser807Cysfs*2	17	1 (M)	No	No
Late truncation after aa 781	c.2504delC	p.Pro835Hisfs*2	17	1 (F)	Yes	Yes [4]
Late truncation after aa 781	c.2477-?_2713 + ?del	p.(Ser833Thrfs*22)	17	2 (F)	No	No
Late truncation after aa 781	c.2564C > G	p.Ser855*	18	2 (F)	Yes	Yes [4]
Late truncation after aa 781	c.2572delC	p.Arg858Alafs*5	18	1 (F)	Yes	Yes [4]
Late truncation after aa 781	c.2635_2636delCT	p.Leu879Glufs*30	18	2 (F)	Yes	Yes [4]
Late truncation after aa 781	c.2711delC	p.Pro904Glnfs*23	18	1 (F)	Yes	Yes [4]
*Mutations not grouped*						
	c.1612A > G	p.Thr538Ala	1	1 (M)	No	No
	c.2684C > T	p.Pro895Leu	18	1 (F)	No	No
	c.65-?_1944 + ?dup	p.(?)	3–12	1 (M)	No	No
	c.146-?_463 + ?dup	p.(?)	5–7	1 (F)	No	No
	(Position uncertain)	p.(?)	1–3	1 (M)	Yes	Yes [4]
	c.745-2A > G	p.(Phe249_Lys275del)	10	1 (F)	Yes	Yes [4]
	c.825 + 1G > A	p.(Phe249_Lys275del)	10	1 (F)	Yes	Yes [4]
	c.825 + 1G > T	p.(Phe249_Lys275del)	10	1 (F)	Yes	No
	c.1030_1031insGAC	p.Lys344delinsArgGln	12	1 (F)	Yes	Yes [4]
	c.2276 + 1G > A	p.(Val718_Trp759delinsGly)	15	1 (F)	Yes	Yes [4]

CDKL5 numbering based on GenBank reference sequences NG_008475.1 and NM_003159.2, with the first A in the start codon numbered +1. Protein names in brackets indicate inferred changes based on *in silico* analyses, including splicing predictions by SpliceSiteFinder-like (accessed through Alamut Visual v2.3.4), MaxEntScan [22], NNSPLICE by the Berkeley Drosophila Genome Project [23], GeneSplicer [24] and Human Splicing Finder [25].

**Table 2:** **Pathogenicity of missense mutations within our current sample of individuals with the CDKL5 disorder**

Nucleotide change (cDNA)	Protein change	SIFT (score 1–0)	MutationTaster (***p***value 0–1)	PolyPhen2 (score 0–1)	Align GVGD (Class C0–C65)
c.38 T > C	p.Phe13Ser	Deleterious (0)	Disease-causing (0.999)	Probably damaging (0.999)	Benign (C0)
c.58G > C	p.Gly20Arg	Deleterious (0)	Disease-causing (1)	Probably damaging (0.985)	Benign (C0)
c.119C > T	p.Ala40Val	Tolerated (0.12)	Disease-causing (0.999)	Probably damaging (0.999)	Benign (C0)
c.191 T > C	p.Leu64Pro	Deleterious (0)	Disease-causing (1)	Probably damaging (1)	Benign (C0)
c.364G > A	p.Ala122Thr	Deleterious (0)	Disease-causing (1)	Probably damaging (0.999)	Likely pathogenic (C55)
c.377G > A	p.Cys126Tyr	Deleterious (0)	Disease-causing (1)	Probably damaging (1)	Pathogenic (C65)
c.395 T > G	p.Val132Gly	Deleterious (0)	Disease-causing (1)	Probably damaging (1)	Pathogenic (C65)
c.428 T > A	p. Ile143Asn	Deleterious (0)	Disease-causing (1)	Possibly damaging (0.9)	Benign (C0)
c.458A > T	p. Asp153Val	Deleterious (0)	Disease-causing (1)	Benign (0.07)	Benign (C0)
c.473G > C	p.Arg158Pro	Deleterious (0)	Disease-causing (1)	Probably damaging (0.999)	Benign (C0)
c.514G > A	p.Val172le	Deleterious (0)	Disease-causing (1)	Probably damaging (0.995)	Benign (C0)
c.526 T > C	p. Trp176Arg	Deleterious (0)	Disease-causing (1)	Probably damaging (0.999)	Benign (C0)
c.532C > T	p.Arg178Trp	Deleterious (0)	Disease-causing (1)	Probably damaging (1)	Benign (C0)
c.536C > T	p. Ser179Phe	Deleterious (0)	Disease-causing (1)	Probably damaging (0.999)	Likely benign (C15)
c.577G > C	p.Asp193His	Deleterious (0)	Disease-causing (1)	Probably damaging (1)	Pathogenic (C65)
c.587C > T	p.Ser196Leu	Deleterious (0)	Disease-causing (1)	Probably damaging (0.976)	Pathogenic (C65)
c.595 T > C	p.Cys199Arg	Deleterious (0)	Disease-causing (1)	Probably damaging (0.999)	Pathogenic (C65)
c.620G > A	p.Gly207Glu	Deleterious (0)	Disease-causing (1)	Probably damaging (1)	Pathogenic (C65)
c.656A > C	p.Gln219Pro	Deleterious (0)	Disease-causing (1)	Probably damaging (0.996)	Pathogenic (C65)
c.680 T > C	p.Leu227Pro	Deleterious (0)	Disease-causing (1)	Probably damaging (0.996)	Pathogenic (C65)
c.2684C > T	p.Pro895Leu	Deleterious (0.03)	Disease causing (1)	Possibly damaging (0.578)	Benign (C0)
c.872G > A	p.Cys291Tyr	Deleterious (0.01)	Disease causing (1)	Probably damaging (0.930)	Benign (C0)
c.215 T > C	p.Ile72Thr	Deleterious (0)	Disease-causing (1)	Possibly damaging (0.578)	Benign (C0)
c.1612A > G	p.Thr538Ala	Deleterious (0.04)	Polymorphism (1)	Benign (0.009)	Benign (C0)
c.526 T > C	p.Trp167Arg	Deleterious (0)	Disease causing (1)	Probably damaging (0.999)	Benign (C0)

CDKL5 numbering based on GenBank reference sequences NG_008475.1 and NM_003159.2, with the first A in the start codon numbered +1. *In silico* predictions are carried out through Alamut Visual (v2.4) using SIFT (scale: 1 = tolerated, 0 = deleterious) [26], MutationTaster (*p* value for prediction confidence (not pathogenicity): 0 = low confidence, 1 = high confidence) [27], PolyPhen2 (HumVar module, scale: 0 = benign, 1 = probably damaging) [28], Align GVGD (scale: C0 = benign, C65 = pathogenic) [29].

### Time-to-event analysis of attainment of developmental milestones

**Figure 2:**
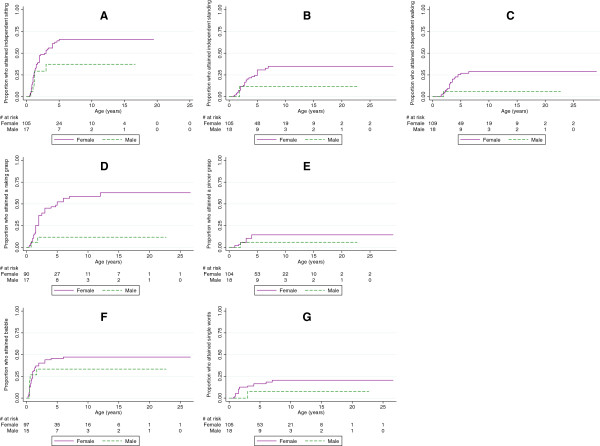
**The proportion of females and males with the CDKL5 disorder who attained developmental milestones by various ages.** Legend: **A**: independent sitting, **B**: independent standing, **C**: independent walking, **D**: raking grasp, **E**: pincer grip, **F**: babble, **G**: single words.

Information on the age of attaining independent sitting was available for all but four females, of whom 59 (56%) had attained independent sitting. The median age at attainment of independent sitting was 3 years (range 6 months to 5 years) with almost three quarters achieving unaided sitting by 5 years of age (Figure 2). Independent standing was attained by 26% of females (*n* = 27/105), and a quarter was able to stand independently by 5 years (range 10 months to 6 years 11 months). Independent walking was attained by 22% of females (*n* = 24/109), and time-to-event analysis showed that a quarter had attained independent walking by 4 and a half years (range 1 year 6 months to 6 years 5 months). For the 18 boys in our study, information on the age of attaining sitting was known for all but one, of whom six could sit independently with a quarter attaining independent sitting by 1 year 3 months (range 8 months to 3 years) (Figure 2). Two boys were able to stand independently by 1 year 10 months, and one boy was able to walk independently at 1 year 11 months.

The age that a raking grasp was attained was known for 92 females of whom 45 (49%) had attained a raking grasp at a median age of 5 years (range 5 months to 12 years) (Figure 2). A pincer grasp was attained by 13% of females (*n* = 14/106) of whom 10% had attained a pincer grip by 3 years (range 1 to 12 years). For the males, information was available for all but one on the age of attaining a raking grasp. Ten percent of the males attained a raking grasp by 1 year 10 months and one male attained a pincer grasp at 2 years of age (Figure 2).

Information on the age that babbling was attained was available for 97 females of whom 43 (44%) had attained babble. A quarter of whom achieved babble by 1 year of age (range 3 months to 6 years) and just under a half by 6 years (Figure 2). Single words were attained by 16% (*n* = 17/105), and 10% would attained single words by 1 year 6 months and just under a quarter by 7 years of age (range 9 months to 7 years). Of the males in our study, information on age of attaining babble was available for all but three (*n* = 15) of whom five attained the ability to babble. A quarter attained babble by 7 months of age (range 6 months to 1 year 8 months) (Figure 2). There was one boy who spoke single words at 3 years of age.

The proportion of females and males who attained other gross motor, fine motor and communication skills not included in the time-to-event analysis is shown in Table 3.

**Table 3:** **The proportion of individuals with the CDKL5 disorder of a particular current age who had attained various gross motor, communication and hand milestones**

	Age at completion of questionnaire							
	1.5 years and under		1.5–7 years		7–13 years		13 years and over	
Developmental skill	Females (***n*** = 11)	Males (***n*** = 1)	Females (***n*** = 50)	Males (***n*** = 10)	Females (***n*** = 32)	Males (***n*** = 5)	Females (***n*** = 16)	Males (***n*** = 2)
*Gross motor*								
Roll front to back	4 (36.4)	1 (100)	46 (92)	5 (50)	27 (84)	2 (40)	14 (88)	1 (50)
Roll back to front	5 (45)	1 (100)	47 (94)	5 (50)	26 (81)	2 (40)	14 (88)	1 (50)
Transition from sitting to crawling position	1 (9)	1 (100)	23 (46)	3 (30)	16 (50)	0 (0)	5 (31)	1 (50)
Crawl	1 (9)	0 (0)	12 (24)	2 (20)	10 (31)	0 (0)	4 (25)	1 (50)
Stand with support	2 (18)	1 (100)	37 (74)	3 (30)	22 (69)	1 (20)	10 (63)	1 (50)
Pull to stand	1 (9)	0 (0)	13 (26)	2 (20)	14 (44)	1 (20)	6 (38)	1 (50)
Walk with support	1 (9)	0 (0)	24 (48)	1 (10)	16 (50)	1 (20)	8 (50)	1 (50)
*Fine motor*								
Transfer from hand to hand	3 (27)	1 (100)	25 (50)	2 (20)	18 (56)	0 (0)	8 (50)	0 (0)
Play with blocks	0 (0)	0 (0)	8 (16)	1 (10)	8 (25)	0 (0)	2 (13)	0 (0)
*Social and communication*								
Social smile	8 (73)	1 (100)	38 (76)	7 (70)	18 (56)	1 (20)	9 (56)	0 (0)^a^
Fix and follow	3 (30.0)^a^	1 (100)	39 (78)	4 (40)	23 (72)	1 (20)	10 (67)^a^	1 (100)^a^
Wave goodbye	0 (0.0)	0 (0)	11 (22)	1 (10)	4 (13)	0 (0)	1 (6)	0 (0)
Respond to own name	4 (36)	1 (100)	33 (66)	3 (33.3)^a^	20 (63)	2 (40)	9 (56)	0 (0)
Point to things that he/she wants	0 (0.0)	0 (0)	6 (12)	1 (10)	8 (26)^a^	0 (0)	2 (13)	0 (0)
Respond to others emotions	4 (36)	1 (100)	23 (47)^a^	4 (40)	11 (34)	1 (20)	4 (25)	1 (50)
Respond to ‘no’	2 (18)	0 (0)	16 (32)	2 (25)^a^	13 (41)	0 (0)	4 (25)	0 (0)
Phrases	0 (0.0)	0 (0)	3 (6)	0 (0)	3 (9)	0 (0)	1 (6)	0 (0)

^a^The denominator in this group varies.

### Relationship between genotype and attainment of developmental milestones for females

**Figure 3:**
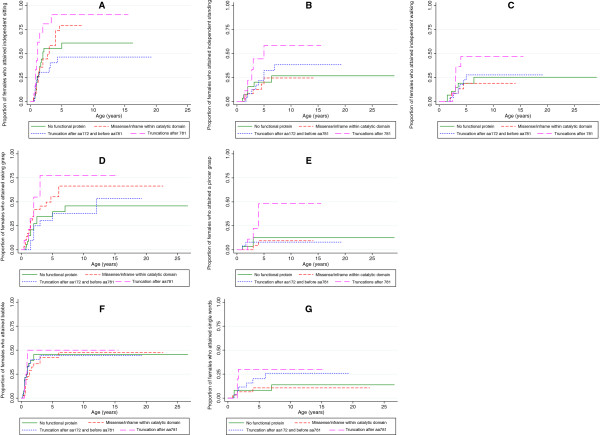
**The influence of mutation type of the proportion of females with the CDKL5 disorder who attained developmental milestones by various ages.** Legend: **A**: independent sitting, **B**: independent standing, **C**: independent walking, **D**: raking grasp, **E**: pincer grip, **F**: babble, **G**: single words.

A higher proportion of females with a truncating mutation after aa 781 attained gross motor, hand function and communication milestones than the other groups (Table 4). Although there was a visible difference between the groups shown in Figure 3, there were no clear relationships, although females with a truncating mutation after aa 781 were more likely to have attained independent sitting than the those with no functional protein (HR: 2.4, *p* value 0.026, 95% CI 1.11–5.36).

**Table 4:** **The relationship between genotype and developmental milestone acquisition in females**

Milestone	No functional protein		Missense/in-frame mutation within catalytic domain		Truncation between aa 172 and aa 781 inclusive		Truncation after aa 781	
	Number (%)	Median age skill attained (years)	Number (%)	Median age skill attained (years)	Number (%)	Median age skill attained (years)	Number (%)	Median age skill attained (years)
Sitting	17/33 (52)	2	18/27 (67)	3	10/26 (38)	-	10/12 (83)	1.2
Standing	6/32 (19)	-	5/28 (18)	-	8/28 (29)	-	5/10 (50)	5
Walking	6/34 (18)	-	4/28 (14)	-	6/28 (21)	-	5/12 (42)	
Babble	13/29 (45)	-	13/27 (48)	6	12/25 (44)	-	4/8 (50)	1
Single words	4/32 (13)	-	3/28 (11)	-	6/28 (21)	-	3/10 (30)	
Raking grasp	12/29 (44)	7	16/25 (64)	4	8/22 (36)	12	7/9 (78)	2
Pincer grasp	3/32 (9)	-	2/28 (7)	-	2/28 (7)	-	4/10 (40)	-

(-) Values blank as less than 50% attained the particular skill.

## Discussion

The acquisition of developmental milestones was severely impaired in our sample of individuals with the CDKL5 disorder. This was especially true for males, with nearly half not achieving any of the major gross motor, communication or hand function milestones investigated. There was variability, with a few girls eventually attaining most if not all of the milestones we investigated. If milestones were attained, they were significantly delayed. Despite small numbers, it appears as though mutation type may play some role in this variation, with girls with a truncating mutation after aa 781 attaining more milestones than those who had a mutation resulting in no functional CDKL5 protein.

Our study has shown that there is variability in the attainment of developmental milestones within the CDKL5 disorder. The majority of females did achieve some milestones, most frequently involving gross motor skills. For most, these milestones were attained at an older age than expected for the general population [30]. Using time-to-event analysis, we found that 10% of females would attain independent sitting by 10 months, by 1 year 2 months a quarter attained independent sitting and by 5 years nearly three quarters had attained independent sitting. In our entire female population, only 22% attained independent walking compared to 31% of our 7- to 13-year group. Time-to-event analysis indicated that 25% of our group attained this skill by 4 and a half years of age. This delay in attainment of milestones occurred across developmental areas and may explain why other studies have found females with the CDKL5 disorder to attain less developmental milestones [3, 7, 31]. In the study by Bahi-Buisson and colleagues, only 1/20 females was able to walk independently, a quarter had reported functional hand use and a quarter could use babble or single words [3]. The increased number of participants in our study compared to the French study is one likely explanation for the differing results. However, age may also be a factor as the median age of the females in the French study (4.5 years) was 2 years less than in ours. We have shown that there is a delay in the age of acquiring these developmental milestones and therefore some of the girls in the French study may be yet to acquire these abilities. There also appears to be a ‘cohort’ effect in our data, with some of our older females (13 years and older) appearing to have attained less developmental milestones than those in the younger age group (7 to 13 years). In the past, children and adults with severe undiagnosed epilepsy disorders were targeted for screening for mutations in the *CDKL5* gene. With advances in genetic technologies, less severely affected children are being tested and identified, thus increasing the variability seen initially in children with the CDKL5 disorder. Regardless, it is important that this variability in functional ability be recognised both by clinicians and researchers.

In our study, the males with the CDKL5 disorder had more impaired development than the females. Again, there was variability, with some of the males having less impaired development. Half of the boys in our study had attained rolling, a quarter learned to sit independently by 1 year 3 months and a quarter used babble by 7 months (earlier than the females in our group). There were four boys in our study who had attained more developmental milestones. The first was a 10-month old who could sit independently, babble and transfer objects from hand to hand (c.400C > T, p.Arg134*). The second was a 2 and a half year old who could stand independently, transfer objects from hand to hand and babbled (c.351 T > A, p.Tyr117*). The third was a 3 and a half year old who could sit independently, had limited hand use and could use single words (c.1612A > G, p.Thr538Ala). The fourth was a 4 and a half year old who had never had epilepsy, could walk independently (even run) and had a pincer grip but had language impairment (babble only) (c.514G > A, p.Val172Ile). There have been few boys with the CDKL5 disorder described in the literature [7, 9, 12, 18, 32–38]. A recent study by Mirzaa and colleagues reported eight males with the CDKL5 disorder whose ages ranged from 2 months to 14 years (median age 5 years) [36]. These boys were described as having profound developmental delay, with impaired or absent language and motor abilities. The authors concluded that boys with the CDKL5 disorder are severely affected and have minimal acquisition of developmental skills. In our study, around half of the males also had minimal acquisitions of developmental skills; however, the other half did attain some milestones. These findings show the variability in the acquisition of developmental milestones in males with the CDKL5 disorder. Although the males are generally more severe than females, more males achieved developmental milestones in our present study than previously reported. Our sample of boys was double that of the previously reported study, and so again increased sample size may be a key factor in being able to demonstrate this variability. Age could also be a factor, however the median ages of the boys in our and the US study [36] were similar (4.3 years and 5 years). It is likely that the previously described ‘cohort effect’ may also be impacting on the variability seen in our male sample. The majority of males in the literature were identified through screening groups of severely affected individuals for *CDKL5* mutations. Advances in genetic technology for diagnostic purposes mean those children who would previously never have been identified, such as our young boy who could run and who had never been diagnosed with epilepsy, are being diagnosed. These findings suggest that a different spectrum of boys with the CDKL5 disorder, who until now have not been diagnosed, may exist.

Our understanding of the functional role of CDKL5 is still limited, but CDKL5 has been shown to be involved in neuronal cell differentiation and proliferation, dendritic arborisation and dendritic spine morphology [39–41]. It is likely that mutations affecting the function of CDKL5 are likely to impair neuronal development and subsequent attainment of developmental milestones. The occurrence of early refractory epilepsy in the CDKL5 disorder may also contribute to the altered neuronal function, with seizures shown to impact developmental programming [42]. Whether the impairment seen in the CDKL5 disorder is a direct result of the occurrence of infantile seizures, resulting in an epileptic encephalopathy, or whether it is a consequence of the underlying gene mutation, is yet to be determined and further research in this area is needed.

The relationship between genotype and phenotype in the CDKL5 disorder has only been specifically investigated in one other study [43]. This study identified 12 patients (from a total of 26 with a mutation) who had one of eight recurrent mutations previously described in the literature. These 12 patients were pooled with an additional 14 individuals previously described in the literature. The authors found that those with the p.Ala40Val, which is a missense mutation within the ATP binding site of the kinase domain, had a milder phenotype (phenotype severity based on factors such as gross motor ability, hand function, stereotypies and seizure severity) than those with a missense mutation elsewhere in the kinase domain or a frameshift within the C-terminal region. Due to the heterogeneity of mutations within the CDKL5 disorder, we chose not to investigate specific mutations, rather, we pooled together different types that were predicted to have similar functional/structural consequences. This heterogeneity means that genotype/phenotype studies will be more difficult to conduct unless larger numbers of individuals are ascertained. We found no clear relationship between genotype and phenotype in our study, but there were a few differences between the groups. Females with a truncation between aa 172 and aa 781 attained fewer developmental milestones, similar to that of those who lacked the entire *CDKL5* gene. Earlier research suggested that loss of the C-terminal region can in fact act as a loss of function mutation due to loss of localisation ability and increased self-phosphorylation (increased capacity to phosphorylate the kinase itself) [16, 44]. One difference we found was that individuals with a truncating mutation after aa 781, such as the mutation c.2635_2636delCT, appeared to attain more milestones overall. This differs from the previous study, which found that girls with this mutation were more severely affected and unable to walk [43]. No clear conclusion can be drawn on the role of genotype until even larger studies are undertaken and other previously identified factors (such as epilepsy) are considered, although it does appear as though genotype may have a part to play in the clinical variability in the CDKL5 disorder.

Our study is the first major international data collection of individuals with the CDKL5 disorder. This gives us the advantage of having the largest data collection on individuals with the CDKL5 disorder. Our data collection tools were also designed to capture clinical information specific to the CDKL5 disorder, not other disorders such as RTT. It is apparent that there is much variability within this disorder, and therefore larger numbers are still needed to provide a more comprehensive representation of the clinical presentation. Recall error, especially for caregivers with older children, is likely. We have limited this by asking caregivers to refer to their child’s medical records and diaries before completing the questionnaire. Because these children exhibit symptoms from an early age, developmental gains are most likely to be well recorded, as caregivers are paying special attention to their child’s development. We found that many parents knew whether their child attained a particular milestone, but the age that these skills were attained was not always known, which is demonstrated by the missing data in our study. Changes in diagnostic technology and increased awareness of the CDKL5 disorder mean that children are being diagnosed younger, and a more variable spectrum is being identified. This results in the ‘cohort effect’ to which we have alluded, where the younger are less severe than previously diagnosed children. The identification of younger children means that this data collection will have the capacity to collect prospective data on developmental milestones. Finally, our study will only reach caregivers of individuals who have access to the internet and therefore selection bias exists in that regard.

## Conclusion

Understanding the acquisition of gross motor milestones in the CDKL5 disorder is valuable information for caregivers, their therapists and clinicians and shows that for most, skills are attained at a much later age. It would be of interest to further investigate the events surrounding the acquisition of these milestones, especially with regard to seizure control. For caregivers, our data provide hope that their young child may still attain milestones at a later age than is usual for the general population. As the database grows, it will be important, to further examine the relationship between genotype and functional abilities. Individuals with the CDKL5 disorder have previously been described as having severely impaired development. Our findings show the variability within this disorder and suggest that there may be children with the CDKL5 disorder who present differently to the clinical picture originally described. Although we found no clear relationship between genotype and phenotype, the differences between individuals suggest that with greater numbers such a relationship may be identified. Continued research into the natural history of the CDKL5 disorder is needed to further our understanding of the variability seen within this condition.

## Abbreviations

CDKL5: cyclin-dependent kinase-like 5
InterRett: International Rett syndrome Phenotype Database
RTT: Rett syndrome
Aa: amino acid.
